# Medically explained symptoms: a mixed methods study of diagnostic, symptom and support experiences of patients with lupus and related systemic autoimmune diseases

**DOI:** 10.1093/rap/rkaa006

**Published:** 2020-02-26

**Authors:** Melanie Sloan, Rupert Harwood, Stephen Sutton, David D’Cruz, Paul Howard, Chris Wincup, James Brimicombe, Caroline Gordon

**Affiliations:** r1 Behavioural Science Group, Institute of Public Health, University of Cambridge, Cambridge; r2 Patient and Public Involvement in lupus Research Group, Institute of Public Health, University of Cambridge, Cambridge; r3 The Louise Coote Lupus Unit, Guy’s and St Thomas’ Hospital, London; r4 LUPUS UK, St James’ House, Romford; r5 Department of Rheumatology, University College London, London; r6 Rheumatology Research Group, Institute of Inflammation and Ageing, College of Medical and Dental Science, University of Birmingham, Birmingham, UK

**Keywords:** systemic lupus erythematosus, patient views, quality of life, patient–physician interaction, misdiagnoses, symptoms, UCTD, diagnostic delays, medical support

## Abstract

**Objectives:**

The aim was to explore patient experiences and views of their symptoms, delays in diagnosis, misdiagnoses and medical support, to identify common experiences, preferences and unmet needs.

**Methods:**

Following a review of LUPUS UK’s online forum, a questionnaire was posted online during December 2018. This was an exploratory mixed methods study, with qualitative data analysed thematically and combined with descriptive and statistically analysed quantitative data.

**Results:**

There were 233 eligible respondents. The mean time to diagnosis from first experiencing symptoms was 6 years 11 months. Seventy-six per cent reported at least one misdiagnosis for symptoms subsequently attributed to their systemic autoimmune rheumatic disease. Mental health/non-organic misdiagnoses constituted 47% of reported misdiagnoses and were indicated to have reduced trust in physicians and to have changed future health-care-seeking behaviour. Perceptions of physician knowledge and listening skills were highly correlated with patient ratings of trust. The symptom burden was high. Fatigue had the greatest impact on activities of daily living, yet the majority reported receiving no support or poor support in managing it. Assessing and treating patients holistically and with empathy was strongly felt to increase diagnostic accuracy and improve medical relationships.

**Conclusion:**

Patient responses indicated that timely diagnosis could be facilitated if physicians had greater knowledge of lupus/related systemic autoimmune diseases and were more amenable to listening to and believing patient reports of their symptoms. Patient priorities included physicians viewing them holistically, with more emotional support and assistance in improving quality of life, especially in relation to fatigue.

**Key messages**

- Diagnostic delays and misdiagnoses are common in SLE/CTD and can damage trust in physicians.
- SLE/CTD patients strongly value physicians taking a holistic view, listening, and believing patient-reported symptoms.
- More support is required with adapting after SLE/CTD diagnosis and in improving quality of life.

## Introduction

SLE is a chronic, inflammatory, autoimmune disease, which can be life threatening. With no definitive diagnostic tests for SLE and related diseases and with a diversity of often non-specific presenting symptoms [1], patients are largely reliant upon expert medical opinion for a diagnosis, with delays in diagnosis and subsequent treatment commonly reported [2–4]. Multiple studies indicate weaknesses in how medical training addresses SLE and other rheumatological conditions [5–8] and highlight a need for greater awareness amongst clinicians in order to aid faster diagnosis [9]. In addition, there have been calls for more patient-focused research [2].

The problem of undiagnosed lupus in the community was recognized >20 years ago [10]. Despite attempts to increase awareness of the disease, more recent studies report lengthy journeys to diagnosis [2–4], with many patients having accrued considerable damage to their health by the time they reach diagnosis and with earlier treatment being associated with better prognosis [11–13]. A 2014 survey of >2500 LUPUS UK members found that the mean time to diagnosis from initial symptom awareness was 6.4 years, with approximately half reporting that they were initially misdiagnosed [2]. Diagnostic delays of >3 years were also reported by 58% of lupus participants in the 2017 Rare Autoimmune Rheumatic Diseases Alliance (RAIRDA) survey [4].

Guidance for UK practitioners was lacking until the publication of the British Society for Rheumatology guideline for the management of lupus in 2017 [14]. Research on patient experiences and preferences is key to increasing the impact of the guideline, because diagnosis and effective disease management is likely to be influenced strongly by patient–physician interactions.

There has been important patient perspective research in recent years, including research reporting on access to health care, diagnostic delays and the frequency of misdiagnoses [2–4, 9]. However, significant gaps in the literature remain, particularly with regard to ascertaining patient views of the reasons for, and impacts of, diagnostic delays and misdiagnoses. In this study, we address these gaps and investigate patient perceptions of their symptoms, support received from clinicians and patient suggestions for improvements in diagnosis and care.

## Methods

### Data collection

After a review of patient priorities on the LUPUS UK Web-based forum and discussions with patients, LUPUS UK and rheumatologists, a questionnaire (supplementary material, section Questionnaire, available at *Rheumatology Advances in Practice* online) was designed to collect quantitative and qualitative data. The main survey sections included: perceptions of support, symptoms, and reasons for misdiagnoses/delays. Open questions to elicit qualitative responses covered: misdiagnosis experiences, advice to doctors on improving diagnosis and care, and an invitation at the end of the questionnaire to give any further patient views. The questionnaire was pre-tested and then made available, with an accompanying information sheet, for 3 weeks in December 2018 for completion online using Qualtrics, on the LUPUS UK online forum and Lupus UK sufferers Facebook group. The supplementary material section Methodology, available at *Rheumatology Advances in Practice* online, provides a more in-depth description of data collection, analysis and study limitations. This study complies with the Declaration of Helsinki. The Cambridge psychology research ethics committee approved the research [PRE 2018–84], and informed consent was obtained from all respondents.

Inclusion criteria were as follows: age ≥18 years; reporting a diagnosis of lupus, UCTD, MCTD, SS or overlap condition on their clinic letters; and listing symptoms that were supportive of these diagnoses, including, in the case of those reporting a diagnosis of SLE, symptoms that were supportive of a diagnosis made in line with the ACR and/or SLICC classification criteria [15, 16]. The 2% reporting probable lupus were also included, because their reported symptoms met the criteria.

### Analysis

After data cleaning and removal of ineligible participants, quantitative data from 233 participants were analysed using SPSS v.25. Qualitative responses were provided by 182 respondents on the questionnaire, with a further 24 providing information by contacting the research team.

Qualitative data were combined and analysed thematically [17] using NVivo v.12 to assist with coding and classification. Briefly, analysis involved: immersion in the data; developing and agreeing an initial coding scheme; coding the data; refining and re-coding; and identifying commonly occurring themes [17]. Once provisional themes had been identified, the LUPUS UK forum moderator (P.H.) posed several questions [18–20] to the online community to confirm that emerging themes reflected the wider community views and to allow the lead researcher (M.S.) to probe responses in more depth. Validity of the findings was also strengthened by member checking [21, 22], comparing emerging themes with forum discussions and examining deviant cases [23]. Further triangulation occurred by the integration of qualitative and quantitative data [24, 25].

## Results

Areas identified from thematic analysis to improve diagnosis and care were as follows: the importance of listening and belief; holistic viewpoint; and knowledge acquisition and transfer.

### Participants

Participant characteristics are shown in Table 1. There were 78% of respondents reporting a diagnosis of SLE, 10% with UCTD, and 12% with other related autoimmune conditions. The vast majority of respondents were white and female, and 70% were resident in England.

**Table 1:** Participant characteristics (*n* = 233)

Characteristic	Number	Percentage (rounded)
Diagnosis on clinic letters		
SLE (may have listed additional diagnosis)	181	78
Undifferentiated or unspecified CTD	23	10
APS or overlap syndrome	8	3
SS	7	3
Discoid or cutaneous lupus	5	2
Probable lupus	5	2
MCTD	4	2
Country of residence		
England	163	70
Scotland	33	14
Wales	20	9
USA/Canada	7	3
Australia	5	2
Mainland Europe	4	2
Northern Ireland	1	<1
Time delay to diagnosis		
<1 year	52	22
1–3 years	42	18
4–9 years	46	20
10+ years	57	24
Unsure or non-quantitative response	30	13
Missing	6	3
Age band (years)		
18–29	18	8
30–39	30	13
40–49	59	25
50–59	69	30
60–69	32	14
70+	7	3
Missing	18	8
Ethnic group		
White	193	83
Mixed	4	2
Asian	2	1
Black or African American	1	<1
Other	1	<1
Missing	32	14
Gender		
Female	199	85
Male	7	3
Prefer not to say	1	<1
Missing	26	11
Education		
None	2	1
GCSE/O level	37	16
A level	55	24
Degree	68	29
Postgraduate	36	15
Missing	35	15
Medications (current or within the last year)		
HCQ	143	72
Oral CSs	96	48
Immunosuppressant (MMF, AZA or MTX)	85	43
Injected CSs	52	26
Biologics	12	6
CSA or tacrolimus	7	4
CYC	6	3
Percentage currently taking at least one of the above medications = 91%		

Note that medication percentages are calculated from those reaching that part of the questionnaire (198 participants).

### Delays and misdiagnoses

The average time to diagnosis from first experiencing lupus/CTD symptoms was 6 years 11 months (mean) and 4 years (median), with 24% of respondents reporting that diagnosis took >10 years and 22% reporting that they were diagnosed within 1 year. At least one misdiagnosis was reported by 76% of respondents, with 47% of all misdiagnoses being of non-organic aetiology, psychological, mental health (MH) or medically unexplained symptoms (MUS), with the combined category referred to henceforth as MH/MUS. Misdiagnoses were most frequent amongst respondents with UCTD, with 95% reporting receiving one or more.

**Fig. 1:**
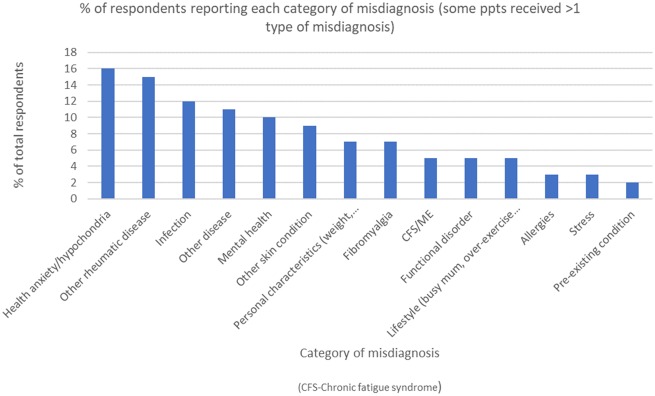
Types and rates of misdiagnoses in order of decreasing frequency *n* = 156 reporting a misdiagnosis from *n* = 205 participants reaching this section of the questionnaire.

Misdiagnoses are summarized in Fig. 1, with health anxiety being the most frequent, followed by misdiagnoses of alternative rheumatic diseases, most commonly RA.

More than 80% of the respondents who reported receiving a MH/MUS misdiagnosis (often referred to by participants and forum members as an ‘in your head’ misdiagnosis) indicated that it had both reduced their future trust in physicians and altered their health-care-seeking behaviour, with the impact of these misdiagnoses illustrated in the following quote:

The misdiagnosis of stress, unexplained etc. is the most dangerous misdiagnosis and it completely destroys trust, feels like you are not being listened to, like you are explaining it wrong, doing something wrong, doing this to yourself and it causes guilt, mistrust and makes you question yourself (female, 30s, England).

The majority (60%) of respondents reported that these MH/MUS misdiagnoses had made them less likely to seek medical help and report symptoms in the future*.* For others (27%), these misdiagnoses made them more likely to seek help, often detailing how they ‘knew’ their ‘own bodies’ and continued to seek clarity for continuing symptoms in the face of medical disbelief in an organic aetiology.

Table 2 summarizes patient views of the key influences on delays and misdiagnoses. The most commonly experienced symptoms before diagnosis were often non-specific (rashes, fatigue, joint pain and oral ulcers) and difficult to attribute to a single disease unless viewed in the context of a systemic illness.

**Table 2:** Participants’ views of reasons for delays/misdiagnoses

Perception of reason for delays/misdiagnosis	Percentage of participants indicating likely/very likely that this had contributed to their delay/misdiagnosis	Example quotes from qualitative responses
The doctor/s initially seeing and treating each symptom separately and not linking together as one disease	80	I used to go in with a long list of medical issues that screamed of APS/lupus/Sjögren’s, but not one GP joined the dots. Sadly, by the time I was diagnosed aged 45 it was too late. I’d had a stroke, DVT [deep vein thrombosis], miscarriages and was struggling to work. I wish I’d been diagnosed earlier and had help so much sooner (female, 50s, Wales)
Symptoms disregarded or disbelieved by the doctor/s (merged with the category of psychological/all in your head misdiagnoses because the two were found largely to duplicate)	72	I feel quite upset that I was diagnosed with ‘just anxiety’ by my GP even to the point where I was lectured on the ‘worried well’ and prescribed a book on health anxiety! I read it diligently but, needless to say, it didn’t make my symptoms disappear (female, 50s, UK)
		I was unsupported for years after a rheumatologist told me my symptoms were all in my mind and had I been sexually abused as a child (female, 50s, England)
The doctor/s not having enough knowledge of the disease (category merged with those feeling they were misdiagnosed owing to not having what doctor/s considered the right test results or typical symptoms)	69	Neurologist said he didn’t believe in lupus and consultant rheumatologist was not interested in discussing symptoms or history as I haven’t lost my hair, didn’t have a malar rash and I’m male. Thankfully, he referred me to a lupus specialist (male, 40s, England*)*
		I was dismissed by rheumatology, having been told ‘there’s nothing wrong’. This was 7 years before diagnosis of SLE by blood test. In that time, I suffered badly, had to reduce working hours and had long periods of being unable to work at all. Symptoms can appear long before markers show in blood (female, 30s, England)
Symptoms appearing slowly over time	59	The symptoms can be confusing; we did not link my previous symptoms together as I just thought I was ageing badly (female, 60s, England)
You not reporting or you underplaying symptoms	39	We underplay our symptoms as we get fed up with constantly moaning about feeling unwell or rubbish (female, 40s, England)

Categories were predetermined from literature review, forum analysis, patient, physician and LUPUS UK input. Five options were given, ranging from very unlikely to very likely, with this section completed only by participants who considered they had experienced delays and/or misdiagnosis (*n* = 175). The seven predetermined questionnaire categories were found to duplicate responses and were therefore condensed into five categories.

Patients reporting atypical serology, especially permanently negative ANA, experienced much greater diagnostic delays (mean delay of 13 years for permanently ANA-negative participants) than ANA-positive patients, with 92% (exact binomial 95% CI: 62%, 100%) of seronegative respondents taking >4 years to be diagnosed, compared with 55% (95% CI: 44%, 66%) with positive ANA and 65% (95% CI: 48%, 78%) with intermittently positive ANA. No seronegative participants were diagnosed within 1 year, compared with 26% of ANA-positive patients (95% CI: 17%, 37%). Using Fisher’s exact test between seronegative, ANA intermittently positive and ANA-positive groups, there was a significant difference (*P* = 0.0386) in the percentages taking >4 years to be diagnosed.

Multiple participants who lacked visible symptoms and/or consistent positive serological markers commented on the sense of invalidation and physician disbelief when test results did not reflect the severity of their symptoms, as articulated by this participant:

The rheumatologist sent me to see a psychiatrist because I complained that I felt ill even though the blood results were OK. The previous results were positive. Psychiatrist said that I was OK and I should get a new rheumatologist who knew more about lupus (female, 50s, England).

Additional evidence of the greater diagnostic/care difficulties experienced by the permanently ANA-negative group was their very high rate of MH/MUS misdiagnoses (92%). However, those reporting positive anti-dsDNA still had a mean diagnostic delay of 6 years 2 months, and 54% reported an MH/MUS misdiagnosis.

### Missed diagnosis and misdiagnoses after diagnosis

A quarter of participants felt that diagnoses provided for new symptoms arising post SLE diagnosis were incorrect. A quarter of these perceived that they were misdiagnosed with fibromyalgia; 25% with anxiety or MH issues; and 8% with functional disorders, either gastrointestinal or neurological; and, as instanced in the following quote, many participants had been subject to multiple misdiagnoses:

Myriad of patronising psychological assumptions, including health fixation disorder, anxiety and fibromyalgia. All while flaring with eventual proven pathology (female, 50s, Australia).

Other participants expressed concern that new symptoms were attributed too quickly to their SLE, with other potential causes not appropriately investigated, as this participant details:

I had to have my gall bladder removed quite suddenly and my GP [general practitioner] had been telling me that lupus caused digestive problems so didn’t investigate it (female, 30s, England).

Conversely, this participant, in common with others, reported feeling that some symptoms were not correctly attributed to the disease, leading to greater anxiety over the possible aetiology:

Not understanding what is happening to your body is distressing and scary. One of the symptoms I have (difficulty swallowing food/choking) has never been connected to lupus, but I am sure this is the case (female, 60s, England).

### Symptoms, serology and disease burden

**Fig. 2:**
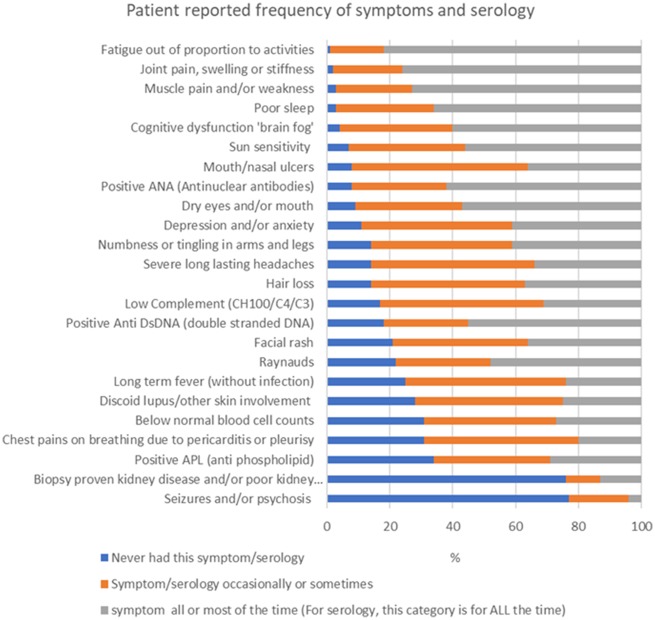
Patient-reported symptoms and serology *n* = 200. Only respondents stating they had a diagnosis of SLE on their clinic letters and meeting ACR and/or SLICC criteria were included in the serology categories (self-reported by those who knew their serological results, e.g. 111 participants for ANA).

As Fig. 2 demonstrates, the symptom burden was high. Fatigue was reported to be present all or most of the time by 82% of respondents and specified by the majority to be the symptom impacting their life the most, followed by pain, then cognitive dysfunction. The invisibility of these symptoms to society and physicians generates an additional challenge of feeling ‘disbelieved’, as highlighted by this respondent:

The fatigue and the pain really is life changing. It’s not easy to see, but it’s there and very real (female, 30s, England).

Interestingly, only 3% of patients responded that organ involvement had the most significant impact, including those with lung, heart, brain or kidney involvement, who predominantly also considered fatigue or pain to be most impactful.

The life-changing impact of these multiple, largely invisible symptoms was highlighted by many, including the reduction in physical and cognitive abilities; greatly reduced quality of life; and the impact on families, social life and employment. The majority (77%) had stopped working, reduced hours or changed jobs at some stage as a result of their disease, with 34% of participants not currently working because of their health issues.

### Support

**Fig. 3:**
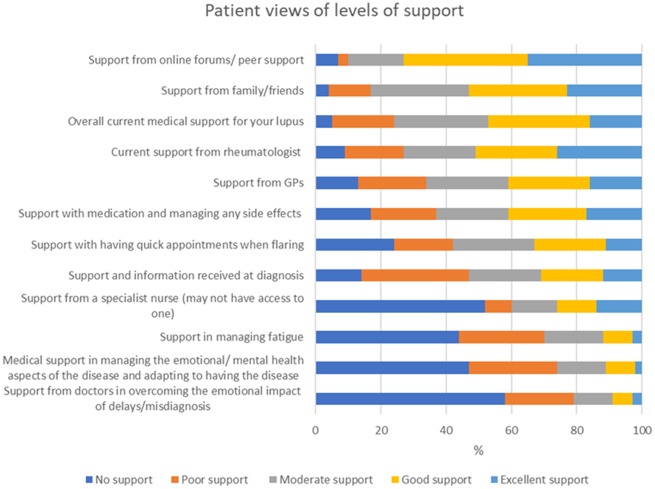
Perceived level of support (*n* = 211) ordered by decreasing level of support

Fig. 3 displays that approximately half of respondents perceived that they were receiving good or excellent support overall and from their rheumatologist. Mean scores (1–5, with 1 being no support to 5 being excellent support) for overall support were less for UCTD (2.6) and patients in Wales (2.7), and slightly higher for anti-dsDNA-positive participants (3.5), than the overall mean (3.3).

The need for much greater support with adapting, accepting and coping mentally with the disease was supported by the qualitative findings and was a key element of the identified holistic viewpoint theme. This theme encompasses the indicated patient belief that physicians should improve diagnosis by viewing symptoms together and should improve treatment by providing care for every aspect of the patient affected by the disease. It was commonly expressed that this care should include more support and empathy; consideration of mental in addition to physical health; and help with improving quality of life and with learning to live with a life-changing disease. Aspects of this theme are reflected in the following participant quote:

I have treatment for the disease but not as a person who has lost so much. It’s a devastating illness.… Delays in diagnosis are damaging physically and mentally. There is a clear pattern of grief, coming to terms with a massive loss of quality of life. I’ve had no emotional support (female, 40s, England).

### Clinician support: knowledge, trust and listening skills

Respondents were asked to rate the different types of clinicians they had consulted for their SLE/CTD for listening skills, knowledge of lupus and the level of trust the patient felt for that type of doctor. The options provided were very poor, poor, moderate, good or very good.

Clinicians consulted by >15% of respondents included (in descending order of quantity consulting) GP, rheumatologist, lupus specialist, accident and emergency (A&E) doctor, specialist nurse, physiotherapist, neurologist, dermatologist, cardiologist, nephrologist, psychologist/counsellor, haematologist and immunologist.

**Fig. 4:**
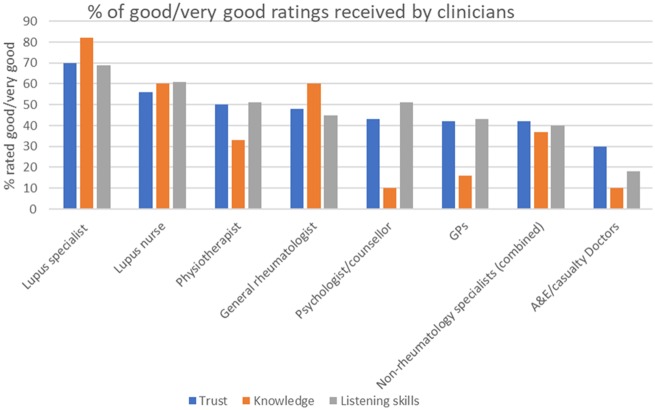
Patient ratings for clinician knowledge, listening skills and trust (*n* = 206) in order of decreasing patient trust

The ratings for non-rheumatology specialists were analysed separately by speciality, but were largely similar so were combined in Fig. 4, with any significant differences reported individually.

The clinicians considered to have the poorest knowledge of lupus were A&E doctors, GPs and neurologists, with respective ratings for poor/very poor knowledge being 66, 50 and 51%.There was a mean of 36% poor/very poor ratings for the knowledge of all specialist physicians, excluding rheumatology clinicians.

Knowledge acquisition and exchange was a major theme identified. Knowledge acquisition by physicians was felt to be of prime importance for diagnosis, whereas knowledge exchange becomes a patient priority post-diagnosis. This theme included patients wanting physicians to provide them with more information, especially at the diagnostic appointment. It also included physicians being more amenable to patients sharing their (often extensive) knowledge of the disease and their individual manifestations. The importance of physicians admitting limited disease-specific knowledge and referring more quickly for appropriate testing and consultations with specialists was also identified within this theme. Many patients reported feeling that MH/MUS misdiagnoses were attributable to lack of knowledge by the physician of immune dysfunction and not listening to/believing a patient’s symptoms.

Trust was highest in lupus specialists, specialist nurses and physiotherapists. For all physician categories, trust exhibited a strongly positive correlation with both perception of listening skills (mean *r* = 0.83) and knowledge (mean *r* = 0.79). The correlation between trust and knowledge was lowest for psychologists (*r* = 0.58) and GPs (*r* = 0.72). Several participants reported that their expectations of knowledge in these clinicians is not high, but that they valued their support and listening skills. One participant, for example, wrote:

I have a very amazing GP; she listens to me each time I see her. I guess it can be harder for GPs to have the full knowledge as they see so many illnesses on a daily basis. For me, just being listened to is a good feeling. My GP cannot fix me but she has really helped me so much in this battle (female, 30s, England).

Most clinicians, including rheumatologists, had ∼30% of ratings in the poor/very poor categories for listening, with the exception of lupus specialists, immunologists and specialist nurses, who received fewer ratings of poor/very poor (16–20%), and A&E doctors, who received the most poor/very poor ratings for listening (56%).

The overarching qualitative theme was the importance of listening and belief in patient symptom reporting, in terms of being essential in linking multiple diverse symptoms for a quicker diagnosis and identifying flares, in validating the subjective experiences of patients and building a supportive relationship with the patient, as explained by this patient:

He (lupus specialist) said he believed everything I was saying and asked me what life is like living with this. It was very emotional for me because I felt listened to (female, 30s, England).

Further sub-themes and patient quotes can be found in Supplementary Table S1, available at *Rheumatology Advances in Practice* online.

## Discussion

This mixed methods study identified a number of commonly occurring patient experiences, both before and after a diagnosis of SLE/CTD. As far as we are aware, this is the first study to ascertain the opinions of SLE/CTD patients regarding causes of delays/misdiagnoses and perceptions of support, including impact upon future health-care behaviour.

With 76% of respondents reporting at least one misdiagnosis and 24% having taken >10 years to reach diagnostic certainty, the diagnostic challenges identified by earlier studies [2–4] remain of key importance to address. Although diagnostic difficulties are attributable, in part, to challenging and varied presentations of the disease, the importance of listening to and believing patients is highlighted by this study, with many patients reporting early symptoms being dismissed and ∼30% of all ratings for clinician’s listening skills being in the poor/very poor categories. Viewing symptoms and patients holistically and physicians acquiring and sharing knowledge were also key areas identified to improve diagnosis and care.

MUS/MH misdiagnoses were reported as damaging to MH, trust in physicians and health-care-seeking behaviour, occurring in almost half of misdiagnosed participants before diagnosis. This propensity for early psychological misdiagnosis in systemic autoimmunity is in line with a recent US study of >3000 SLE patients in which >50% were initially told there was nothing wrong or their symptoms were psychological [3]. Misdiagnoses were also reported to occur after diagnosis with SLE/overlap disease in 25% of our respondents; these were largely non-organic pain, fatigue or MH related co-diagnoses, which respondents and forum members generally felt were caused by and should be attributed to their CTD.

Our findings highlight that fatigue is a prevalent and debilitating symptom, in keeping with a number of previous studies [2, 9, 26, 27], and remains a problem even when the disease is not obviously clinically or serologically active [28]. Despite limited understanding or consensus on mechanisms and an absence of proven biomarkers [29], fatigue is clearly a major unmet need, with >70% of participants reporting no support or poor support with managing fatigue in our study and others [30]. This affects those with UCTD or non-organ involvement SLE as much as those with severe SLE.

In terms of between-groups differences, participants with UCTD and participants resident in Wales had the poorest perception of overall medical support. Great concerns over local barriers to accessing specialist care were detailed by those living in Wales.

The first physicians likely to be assessing these patients are those rated by >50% of patients as having very poor/poor knowledge of SLE (predominantly GPs and A&E clinicians), with correct testing, referrals to specialists and treatment often reported to be delayed. Many of the patients in this study were disadvantaged by a presentation of multiple symptoms, multiple systems, multiple times often being attributed to MH/MUS, especially if there are no presenting visible symptoms or organ involvement. Tschudi-Madsen *et al.* [31] reported that the hypothesis prevalent in current literature is that multisymptomatology and MUS are closely related and found a strong correlation between physicians’ assessment of MUS and the number of symptoms reported. Although it can be difficult for a non-specialist to identify lupus, and investigating the underlying cause of symptoms is often delayed by logistical and time constraints in primary care and by stringent criteria for referrals, multiple diverse symptoms should also be considered a red flag for considering an autoimmune multi-system disease.

One of the barriers to diagnosis is from physicians mistakenly considering ANA and anti-dsDNA positivity as essential for referral to rheumatology/diagnosis, also sometimes failing to interpret or test for other relevant haematological or immunological abnormalities, as specified in the BSR and other guidelines [14–16]. Although self-reported in this study, the 8% of seronegative and the 32% of SLE patients with a combination of historical positive and negative ANA results is in line with expert opinion and recent research findings. Autoantibody profiles can change over time, can vary in titre and positivity independent of disease activity, are heavily influenced by which test individual laboratories use, can become negative over time/owing to medication, and symptoms may occur before development of autoantibodies [32–36]. Although true seronegativity (permanently negative ANA and anti-dsDNA) is often estimated in 5–8% of the SLE population, this includes only those who have achieved diagnosis. The much greater diagnostic delay in seronegative patients in this study (>4 years for 92% compared with 55% for seropositivity and 65% for intermittent ANA positivity) was statistically significant (*P* = 0.0386) and suggests that the real percentage is much higher, with many potentially remaining undiagnosed and untreated.

Despite anti-dsDNA-positive participants having the most specific biomarker for SLE, they still experienced considerable diagnostic delay, with a mean time to diagnosis of 6 years and 2 months, and with 54% reporting having received an MUS/MH misdiagnosis. Although these figures were better than those for permanently seronegative participants (for whom the mean time to diagnosis was 13 years, and 92% of whom reported an MH/MUS misdiagnosis), they still raise the question of whether there is sufficient knowledge and early testing of autoantibodies in primary care.

Multiple respondents reported, in common with other studies, feeling disbelieved when typical serological/other test results did not reflect their symptoms. There is a perceived over-reliance on certain test results, rather than also considering subjective symptoms [37, 38], and a need for more widespread use and understanding of a broader range of haematological and immunological tests [14]. This study also highlights the need for more support, empathy and communication in managing all symptoms, regardless of serology or (often conflicting and still evolving) views of their aetiology.

Although ∼50% of respondents reported either good or excellent support for their lupus overall and from their rheumatologist, there were common gaps in care identified. There is a clearly expressed need for more support for patients in coming to terms with a life-changing chronic disease and more consideration of psychosocial needs and quality of life. Empathetic listening and belief in the patient’s subjective reporting of symptoms was highlighted as the top patient priority for improvements for diagnosis and care, yet this aspect of consultations was often felt to be unsatisfactory in this study and other studies of rheumatic diseases [39]. Listening was highly correlated with trust, with positive views being expressed about physicians, often GPs, who provided support and compassion despite limited knowledge and diagnostic uncertainty.

The main limitation of this study was that participants were not demographically representative of the SLE population, especially in terms of ethnicity, with patients of African and Asian origin under-represented, as is common in rheumatological research [40]. Online support groups may also not be representative of all patients in terms of age, education, severity of disease and satisfaction with medical support because they are actively seeking more peer support. We will address these concerns with a prospective clinic-based study in the future. Although the research and questionnaire were phrased neutrally, the study might have attracted a higher proportion of patients with negative experiences. The responses, including previous symptoms and serological results, might be subject to recall bias and error owing to self-reporting. The serological results might be skewed towards those with abnormal results because these will have been more likely to be communicated to patients and be more memorable. Mean diagnostic time and proportion in the >10 years to diagnosis group are likely to be greater than reported because >10% of responses were excluded owing to non-numerical responses, usually for lengthier time scales (e.g. ‘a long time’).The assessment of misdiagnoses was from the viewpoint of the participants. Some respondents might have had a concurrent MH condition alongside SLE, or MH symptoms might have been a presenting part of their unrecognized developing systemic autoimmunity.

Although a limitation is that diagnoses were self-verified, we mitigated against this by asking for the diagnosis on their clinic letters and checking that symptom lists were indicative of these conditions. A further check was that 91% of participants were currently taking at least one medication prescribed to manage SLE and related conditions. Responses on a questionnaire are less likely to be subject to social desirability bias and more open than responses given to physician questioning, especially regarding the reporting of negative views about medical care. Analysis of both quantitative and qualitative data allows for greater depth and breadth of understanding [24, 25] and increases the validity, reliability and credibility through the triangulation of results.

The large number of respondents sending additional qualitative information and expressing gratitude for a study that gave them the opportunity to detail their experiences and opinions demonstrates the importance of giving patients with chronic diseases a voice. In addition to the research alerting policy-makers and physicians to common, and possibly avoidable, negative experiences, whilst gaining insight into best practice, the further benefits are that many of these patients reported feeling more empowered and hopeful of improvements in diagnosis and care for patients in the future from being involved in the research.

In conclusion, physicians viewing symptoms/patients holistically and listening to (and believing) patients’ reports of their symptoms is felt to be of prime importance by patients both in achieving diagnosis and in managing the disease post-diagnosis. More medical support could help to improve the reduced quality of life, especially in relationship to fatigue, where the majority of participants reported receiving no support or poor support. Improved knowledge of SLE amongst GPs and all physicians, regardless of specialism, is required because the multi-system nature of the disease necessitates a multi-disciplinary approach.

## Supplementary Material

rkaa006_Supplementary_Data
