# Supplementary material for: Medically explained symptoms: a mixed methods study of diagnostic, symptom and support experiences of patients with lupus and related systemic autoimmune diseases
Source: Rheumatol Adv Pract. 2020 Feb 26;4(1):rkaa006. doi: 10.1093/rap/rkaa006 (PMC7197794; doi:10.1093/rap/rkaa006)
Supplement: rkaa006_Supplementary_Data [file rkaa006_supplementary_data.docx]

**SUPPLEMENTARY MATERIAL**

**Questionnaire**


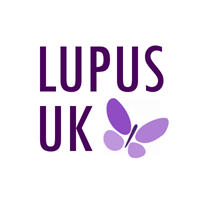

**QUESTIONNAIRE – Your journey to diagnosis and level of medical support**

Thank you very much for completing this questionnaire. Your experiences are very important in helping support people with lupus and related connective tissue diseases. This information will help us to find common patterns, look for ways to improve the situation and raise awareness by ensuring people with these diseases have their experiences understood by researchers, clinicians and decision makers.

**Please note that although the term ‘lupus’ is used for convenience throughout, we are equally interested in the opinions of those of you with overlap diseases such as Sjögrens undifferentiated or mixed connective tissue disease** (UCTD/MCTD) This questionnaire should take about 20 minutes to complete. If you are not feeling well enough and finding it difficult to complete the full questionnaire but are keen to share your views, please just complete what you can or email/ call Melanie using the contact details on the consent form.

**If you wish to clarify any of your answers or give more information, we would be very happy for you to add additional information on a separate sheet or email extra details.** For example, you may wish to write about how supported/unsupported you feel, your feelings about any delays in diagnosis or misdiagnoses, the impact on your relationships and behaviour with doctors and any positive or negative experiences with doctors.

We will be contacting a proportion of people with a range of experiences for an in-depth interview about these topics. **All responses from the questionnaire and any interviews will be anonymised and only the research team will see your personal details.**

| **Name: Country of residence:** |
| --- |
| **Please give your preferred contact details or leave blank if you do not wish to be contacted**  **Email: Phone:** |

| **Section 1 – Your diagnosis** | | | | | | | |
| --- | --- | --- | --- | --- | --- | --- | --- |
| 1. What is the main diagnosis/ diagnoses written on your clinic letters? |  | | | | | | |
| 2. What year/s were you diagnosed? |  | | | | | | |
| 3. How long before your diagnosis was it that you experienced your initial lupus symptom/s? |  | | | | | | |
| 4. Approximately how many times did you see a doctor with lupus related symptoms before diagnosis? |  | | | | | | |
| 5. Which type of doctor diagnosed you? (rheumatologist, GP, dermatologist etc) | **Did you see this doctor:**  Privately/ on the NHS (please circle) | | | | | | |
| 6. Did you seek a second or further opinions on diagnosis after seeing a first rheumatologist? | **Please circle If YES, were further opinions:**  YES/ NO NHS/ Private/ a mixture | | | | | | |
| 7. Have you ever requested a referral or testing for your lupus symptoms and had it refused? | **Please circle**  YES/ NO | | | | | | |
| **Section 2 – Your medical support** | | | | | | | |
| **8. Please fill in the following table about the level of medical support you feel you received/ are receiving** | | | | | | | |
| **Type of support** | | Please circle the number or N/A  **1= No support, 2 = poor support, 3 =moderate support, 4= good support, 5= Excellent support** | | | | | |
| Overall current medical support for your lupus | | 1 | 2 | 3 | 4 | 5 |  |
| Current support by your rheumatologist | | 1 | 2 | 3 | 4 | 5 | N/A |
| Support from a specialised nurse | | 1 | 2 | 3 | 4 | 5 | N/A |
| Overall support by your GP/s | | 1 | 2 | 3 | 4 | 5 |  |
| Support in having quick appointments when flaring | | 1 | 2 | 3 | 4 | 5 | N/A |
| Support with managing medication and any side effects | | 1 | 2 | 3 | 4 | 5 | N/A |
| Support in managing fatigue | | 1 | 2 | 3 | 4 | 5 | N/A |
| Support and information received at diagnosis | | 1 | 2 | 3 | 4 | 5 | N/A |
| Level of medical support in managing the emotional/mental health aspects of the disease and adapting to life with lupus | | 1 | 2 | 3 | 4 | 5 |  |
| Level of support from Doctors in overcoming the emotional impact of any misdiagnoses/ delay in diagnosis | | 1 | 2 | 3 | 4 | 5 | N/A |
| Support from forums and/or lupus support groups | | 1 | 2 | 3 | 4 | 5 | N/A |
| Support from family/ friends | | 1 | 2 | 3 | 4 | 5 |  |

| **9. Please fill in the table below about the clinicians you see/ have seen for your lupus.**  **Please leave the row blank if you’ve never seen this type of doctor for your lupus.** | | | | | | | | | | | | | | | |
| --- | --- | --- | --- | --- | --- | --- | --- | --- | --- | --- | --- | --- | --- | --- | --- |
| **Type of Doctor** | What is your level of trust in these doctors? Please circle one: **1=very poor, 2= poor, 3=moderate, 4=good, 5=very good** | | | | | How do you feel their level of knowledge about lupus is? Please circle one: **1=Very poor 2=poor, 3= moderate, 4= good, 5 = very good** | | | | | How are their listening skills in relation to your symptoms? Please circle one: **1=Very poor 2= poor, 3-=moderate, 4= good, 5 =very good** | | | | |
| GPs | 1 | 2 | 3 | 4 | 5 | 1 | 2 | 3 | 4 | 5 | 1 | 2 | 3 | 4 | 5 |
| Lupus specialist | 1 | 2 | 3 | 4 | 5 | 1 | 2 | 3 | 4 | 5 | 1 | 2 | 3 | 4 | 5 |
| General rheumatologist | 1 | 2 | 3 | 4 | 5 | 1 | 2 | 3 | 4 | 5 | 1 | 2 | 3 | 4 | 5 |
| Specialist Nurse | 1 | 2 | 3 | 4 | 5 | 1 | 2 | 3 | 4 | 5 | 1 | 2 | 3 | 4 | 5 |
| Nephrologist | 1 | 2 | 3 | 4 | 5 | 1 | 2 | 3 | 4 | 5 | 1 | 2 | 3 | 4 | 5 |
| Dermatologist | 1 | 2 | 3 | 4 | 5 | 1 | 2 | 3 | 4 | 5 | 1 | 2 | 3 | 4 | 5 |
| Neurologist | 1 | 2 | 3 | 4 | 5 | 1 | 2 | 3 | 4 | 5 | 1 | 2 | 3 | 4 | 5 |
| Cardiologist | 1 | 2 | 3 | 4 | 5 | 1 | 2 | 3 | 4 | 5 | 1 | 2 | 3 | 4 | 5 |
| Immunologist | 1 | 2 | 3 | 4 | 5 | 1 | 2 | 3 | 4 | 5 | 1 | 2 | 3 | 4 | 5 |
| Haematologist | 1 | 2 | 3 | 4 | 5 | 1 | 2 | 3 | 4 | 5 | 1 | 2 | 3 | 4 | 5 |
| Physiotherapist | 1 | 2 | 3 | 4 | 5 | 1 | 2 | 3 | 4 | 5 | 1 | 2 | 3 | 4 | 5 |
| Psychologist/counsellor | 1 | 2 | 3 | 4 | 5 | 1 | 2 | 3 | 4 | 5 | 1 | 2 | 3 | 4 | 5 |
| A&E/ casualty doctors | 1 | 2 | 3 | 4 | 5 | 1 | 2 | 3 | 4 | 5 | 1 | 2 | 3 | 4 | 5 |
| Other – please write speciality and complete chart |  | | | | |  | | | | |  | | | | |
|  | 1 | 2 | 3 | 4 | 5 | 1 | 2 | 3 | 4 | 5 | 1 | 2 | 3 | 4 | 5 |
|  | 1 | 2 | 3 | 4 | 5 | 1 | 2 | 3 | 4 | 5 | 1 | 2 | 3 | 4 | 5 |

| **Section 3– You and your lupus** | | | | | | |
| --- | --- | --- | --- | --- | --- | --- |
| 10. How old are you? |  | | | | | |
| 11. Gender? | **Please circle one**  Male/ Female/ other/ prefer to not say | | | | | |
| 12. Ethnic background? | **Please circle one**  Asian/ White/ Black/ Chinese/Mixed/ Other | | | | | |
| 13. Please write the highest type of qualification received.  (eg GCSE/O level, A level, NVQ, degree etc) |  | | | | | |
| 14. Please circle the category that best describes your job status. | **Please circle one**  Working full time, Working part time, Student, Retired, Not currently working by choice, Not currently working due to health | | | | | |
| 15. Have you ever had to stop working/ reduce hours or change jobs because of your lupus? | **Please circle one**  Yes/No | | | | | |
|  | | | | | | |
| **16. Please complete the following with how often you experience each symptom. Please put a cross in the final column if you had that symptom before your official diagnosis** | | | | | | |
| **Symptoms/tests results** | **Please circle one**  1= Never had this symptom, 2= occasionally, 3= sometimes, 4 = Most of the time, 5 = Always. | | | | | Put a cross if you had the symptom before diagnosis |
| Facial rash | 1 | 2 | 3 | 4 | 5 |  |
| Discoid lupus/ other skin involvement or rashes | 1 | 2 | 3 | 4 | 5 |  |
| Mouth/nose ulcers | 1 | 2 | 3 | 4 | 5 |  |
| Hair loss | 1 | 2 | 3 | 4 | 5 |  |
| Long- term fevers (without infection) | 1 | 2 | 3 | 4 | 5 |  |
| Sensitive to the sun/lights (get a rash or worsening of symptoms) | 1 | 2 | 3 | 4 | 5 |  |
| Brain fog (memory, attention and concentration difficulties etc) | 1 | 2 | 3 | 4 | 5 |  |
| Fatigue out of proportion to activities | 1 | 2 | 3 | 4 | 5 |  |
| Joint pain, swelling or stiffness | 1 | 2 | 3 | 4 | 5 |  |
| Muscle pain and/or weakness | 1 | 2 | 3 | 4 | 5 |  |
| Seizures (fits) or psychosis (strange thoughts/hallucinations) | 1 | 2 | 3 | 4 | 5 |  |
| Severe long-lasting headaches | 1 | 2 | 3 | 4 | 5 |  |
| Depression and/or anxiety | 1 | 2 | 3 | 4 | 5 |  |
| Numbness or tingling in arms and legs | 1 | 2 | 3 | 4 | 5 |  |
| Chest pains on deep breathing due to Pericarditis or pleurisy (inflammation of lining of heart/lungs) | 1 | 2 | 3 | 4 | 5 |  |
| Biopsy proven kidney disease and/or poor kidney function | 1 | 2 | 3 | 4 | 5 |  |
| Below normal blood cell count (platelets, red, white blood cells) | 1 | 2 | 3 | 4 | 5 |  |
| Poor sleep | 1 | 2 | 3 | 4 | 5 |  |
| Dry eyes and/or mouth | 1 | 2 | 3 | 4 | 5 |  |
| Raynauds (fingers/toes that go white or blue in the cold) | 1 | 2 | 3 | 4 | 5 |  |
| Please write up to 3 of your other main symptoms below and complete the table |  | | | | |  |
|  | 1 | 2 | 3 | 4 | 5 |  |
|  | 1 | 2 | 3 | 4 | 5 |  |
|  | 1 | 2 | 3 | 4 | 5 |  |
| What symptom do you find impacts your life the most? | | | | | | |

| **17.Please circle the number in the table for any of your autoantibody and complement test results you know** | | | | |
| --- | --- | --- | --- | --- |
| **Autoantibody type/ Complement** | **Results: 1 = Always 2= sometimes 3= never** | | | |
| Positive ANA (antinuclear antibodies) | 1 | 2 | 3 | Don’t know |
| Positive ENA (extractable nuclear antigens including anti-Ro, La, RNP and Sm) | 1 | 2 | 3 | Don’t know |
| Positive dsDNA (anti double stranded DNA) | 1 | 2 | 3 | Don’t know |
| Positive APL (antiphospholipid/ anticardiolipin/ lupus anticoagulant) | 1 | 2 | 3 | Don’t know |
| Low complement (C3, C4 or CH100/CH50) | 1 | 2 | 3 | Don’t know |

| **Section 4 – Any Delays/Misdiagnoses.**  Please go straight to section 5 if you feel you did not have any delay in diagnosis or any misdiagnoses | | | | | | | | |
| --- | --- | --- | --- | --- | --- | --- | --- | --- |
| 18. Please list any misdiagnoses you feel you have received for your symptoms **BEFORE** your diagnosis and which type of doctor (GP, rheumatologist, neurologist etc) each one was from: | | | | | | | | |
| 19. Please list any misdiagnoses you feel you have received for your lupus symptoms **AFTER** your diagnosis: | | | | | | | | |
| 20. If you experienced delays/misdiagnoses, please answer the following questions as to how much you feel each one increased the likelihood of delay/misdiagnoses in your case.  **Please circle from: 1= very unlikely, 2= unlikely, 3=possibly, 4=likely, 5= very likely** | | | | | | | | |
| Symptoms disregarded or disbelieved by the doctor/s | 1 | 2 | | 3 | 4 | | 5 | |
| The doctor/s initially seeing and treating each symptom separately and not linking together as one disease | 1 | 2 | | 3 | 4 | | 5 | |
| Symptoms appearing slowly over time | 1 | 2 | | 3 | 4 | | 5 | |
| You not reporting or underplaying symptoms | 1 | 2 | | 3 | 4 | | 5 | |
| Not having what the doctor/s considered to be the ‘right’ lupus blood test results and/or ‘typical’ lupus symptoms | 1 | 2 | | 3 | 4 | | 5 | |
| The doctor/s thinking your lupus symptoms were psychological, mental health problems, functional or ‘all in your head’ | 1 | 2 | | 3 | 4 | | 5 | |
| Not feeling you had a relationship with your doctor/s where you worked together as a team to find the source of your symptoms | 1 | 2 | | 3 | 4 | | 5 | |
| The doctor/s not having enough knowledge of the disease | 1 | 2 | | 3 | 4 | | 5 | |
| **21. If you have ever felt that you received a psychological/psychosomatic misdiagnosis (for example, anxiety, stress, ‘all in your head’, medically unexplained, depression etc) for your lupus symptoms, please answer the following questions: Please circle one answer for each of the questions.** | | | | | | | | |
| How did a psychological misdiagnosis feel compared to being misdiagnosed with a different disease? | No different | | Worse | | | Better | |  |
| How did it change your likelihood to seek help and report your symptoms in the future? | No change | | Less likely | | | More likely | |  |
| How did it change your overall trust in doctors in the future? | No change | | Less trust | | | More trust | |  |

|  | **Section 5 – Your lupus medications** | | | | | | | | | | |
| --- | --- | --- | --- | --- | --- | --- | --- | --- | --- | --- | --- |
|  | **22. Please fill in this table about any MAIN medications you have been prescribed for your lupus (do not include painkillers, vitamins, bone/stomach protection etc.)** | | | | | | | | | | |
| **Medication** | | Please circle one **1=currently taking, 2= Taken in past year, 3 =Taken more than a year ago, 4= never taken** | | |  | Please circle for whether you take/took it exactly as prescribed  **1-=Never, 2= Occasionally, 3= Sometimes, 4=Usually, 5= Always** | | | | | Please put a cross if you had a bad reaction to this drug and couldn’t keep taking it |
| Hydroxychloroquine | | 1 | 2 | 3 | 4 | 1 | 2 | 3 | 4 | 5 |  |
| Oral Steroids (prednisolone, cortisone etc) | | 1 | 2 | 3 | 4 | 1 | 2 | 3 | 4 | 5 |  |
| Mycophenolate (MMF) | | 1 | 2 | 3 | 4 | 1 | 2 | 3 | 4 | 5 |  |
| Azathioprine | | 1 | 2 | 3 | 4 | 1 | 2 | 3 | 4 | 5 |  |
| Methotrexate | | 1 | 2 | 3 | 4 | 1 | 2 | 3 | 4 | 5 |  |
| Ciclosporin or tacrolimus | | 1 | 2 | 3 | 4 | 1 | 2 | 3 | 4 | 5 |  |
| Steroid injections | | 1 | 2 | 3 | 4 | N/A as not self-administered | | | | |  |
| Cyclophosphamide | | 1 | 2 | 3 | 4 | N/A as by infusion | | | | |  |
| Biological (eg rituximab, belimumab) | | 1 | 2 | 3 | 4 | N/A as by infusion | | | | |  |
|  | **Please write any other MAIN medications and fill in the table** | | | | | | | | | | |
|  | | 1 | 2 | 3 |  | 1 | 2 | 3 | 4 | 5 |  |
|  | | 1 | 2 | 3 |  | 1 | 2 | 3 | 4 | 5 |  |
|  | | 1 | 2 | 3 |  | 1 | 2 | 3 | 4 | 5 |  |
|  | 23a. Please give any reasons (for example, forget, don’t like the side effects etc) for NOT taking your medication as prescribed. | | | | | | | | | | |
|  | 23b. Please give any reasons (for example, fear of getting worse, respect for Dr etc) FOR taking your medication as prescribed. | | | | | | | | | | |
| 24. Do you tell your doctor when you haven’t taken the medications as prescribed? | | | | |  | **Please circle one**  Always/ Usually/ Sometimes/ Occasionally/ Never | | | | | |
|  | 25. If you don’t always tell your Dr when you don’t take your medicine as prescribed, please list the reasons why not: | | | | | | | | | | |

| **Section 6- Ideas for improvements** | | | | | | |
| --- | --- | --- | --- | --- | --- | --- |
| 26. If you could give one piece of advice to doctors that you feel would help a quicker diagnosis/ avoid misdiagnoses in lupus/related CTD patients, what would it be? | | | | | | |
| 27. If you could give one piece of advice to doctors caring for patients with lupus what would it be? | | | | | | |
| 28. Please circle the numbers below with how helpful you feel these suggestions would be/would have been for YOU in coping with the disease.  **1= Not at all helpful, 2=slightly helpful, 3= moderately, 4= very helpful, 5 = extremely helpful**  Please put a cross in the final column if you have already had/used this help. Please add any other suggestions you feel would help on an extra page. | | | | | | |
| Receiving a full information pack at diagnosis | 1 | 2 | 3 | 4 | 5 |  |
| Receiving counselling sessions on adapting to and coping with a chronic disease | 1 | 2 | 3 | 4 | 5 |  |
| Doctors receiving more information and training in how to diagnose and help you manage the disease | 1 | 2 | 3 | 4 | 5 |  |
| Receiving regular text messages from Drs, nurses and fellow patients with support and advice | 1 | 2 | 3 | 4 | 5 |  |
| Attending support groups and participating in online forums | 1 | 2 | 3 | 4 | 5 |  |
| Attending a course with other patients where Drs, physios, exercise and diet specialists etc provide information and support to help you manage the symptoms | 1 | 2 | 3 | 4 | 5 |  |

Please return this questionnaire **WITH THE COMPLETED CONSENT FORM** in the envelope provided or online using Qualtrics or by email to [mas229@medschl.cams.ac.uk](mailto:mas229@medschl.cams.ac.uk). We aim to send an updated version of this questionnaire to more people, so if you have any questions you think should be included or if anything didn’t make sense please do let us know.

**Please do add any additional information or clarification on your answers as this will help us understand the patient experience even more.**

**Thank you very much for completing this questionnaire ☺**

**Methodology**

**Data Collection**

Prior to designing the questionnaire, patient representatives and researchers spent 6 months observing and analysing current and past posts/responses on the LUPUS UK forum for common topics of conversation and key concerns. The questionnaire was then designed with a group of 5 patients, LUPUS UK, researcher, psychology and rheumatology input, in order to reflect the observed patient priorities and elicit quantitative and qualitative information on the key areas that concern these patients. The draft questionnaire was pre-tested by 4 of the study patient representatives and academic staff, then by an additional 8 patients for approval/additions/any difficulties in understanding questions. The improved version was approved by the Cambridge psychology research ethics committee.

Prior to the questionnaire being made available, the information sheet was posted online with the opportunity to ask the researchers and group moderators questions. The questionnaire link was then posted, with an accompanying information sheet, for 3 weeks in December 2018 for completion online using Qualtrics, on the LUPUS UK online forum (2,649 members active during Nov 18- Jan 19 – posting or responding at least once) and ‘Lupus UK sufferers’ Facebook group (over 5000 members). Potential participants were given the opportunity to also complete by post or phone, with only one not completing online and opting to complete on the phone. Consent statements were at the start of the questionnaire. These were mandatory to complete and electronically sign before progressing to the questionnaire.

Diagnoses were self-verified. To reduce the likelihood of undiagnosed respondents participating, they were asked to include the diagnoses on their clinic letters. Further clarification was obtained by ensuring listed symptoms were supportive of these diagnoses; including, in the case of those reporting a diagnosis of SLE, symptoms that were supportive of a diagnosis made in line with the ACR and/or SLICC classification criteria. The fact that 91% of participants reaching the medication section of the questionnaire reported currently taking one or more of the listed medications commonly used in the treatment of SLE/ related CTDs is additional evidence that the vast majority of respondents were likely to be currently officially diagnosed with one of these conditions (and of the 9% not currently taking SLE medication, some had taken these medications in the past and stopped due to adverse reactions, and some were newly diagnosed and awaiting to commence treatment – only 5% had never received any of these medications). The evolving nature of these diseases and difficulties in diagnosis may have led to a minority of participants being included who were then undiagnosed or received a change in diagnosis following the study. However, any possible incorrect reporting of a diagnosis/ inclusion of currently misdiagnosed participants is unlikely to have occurred in a sufficient number of cases to have had a significant impact on the overall findings.

**Analysis**

Following data cleaning and removal of (6) ineligible participants, quantitative data from 233 participants were analysed using SPSS version 25 and Excel. Reported proportions and analysis were based upon responses for each individual questionnaire section; with there being approximately 200 participants for the majority of sections. Data presentation was largely graphical and descriptive to ensure accessibility to a wide audience. Correlations were calculated using Pearson’s product-moment correlation coefficient. Fisher’s Exact test was used for determining the statistical significance of differences in diagnostic delay in seronegative, intermittently positive and permanently ANA positive participants.

Qualitative responses were provided by 182 respondents on the questionnaire, with a further 24 providing information by contacting the research team. These additional responses allowed for further discussion and questioning, largely by email conversations, and were included in the generation of initial ideas for emerging themes. Data and quotes were only used from those who had fully completed the online informed consent form. Whilst mixed methodology traditionally incorporates in-depth interviews, the lengthy, detailed responses to the open-ended written questions were sufficiently insightful to allow for the in-depth interviews to be published separately, and also used for triangulation to strengthen validity of this study.

The open-ended questions generated a large amount of insightful data, allowing for the responses to the questions to be combined, coded (categorised) then emerging themes to be discussed and clarified. Analysis was thematic with NVivo 12 used to assist with coding and classification. The stages of analysis involved 1) immersion in the data (repeatedly reading and making notes on the responses) 2) developing and agreeing an initial coding scheme in order to categorise the data to make it more manageable and clear (these included codes such as ‘emotional responses to misdiagnoses’, ‘positive relationships with clinicians’ etc) 3) coding the data (each section of a participant’s responses were assigned to one -or often multiple- codes) 4) refining and re-coding (during coding, some codes were redundant and others were combined) 5) identifying commonly occurring themes^17^ This stage involved individual responses and codes containing data from multiple participants being analysed to identify themes (for example the code for ‘negative relationships with clinicians’ contained multiple responses on disbelief of symptoms and a perception of not being listened to, as did several other codes so ‘listening and belief’ became clear themes arising from the data). The stages involved a regular return to earlier stages to ensure full familiarity with both the raw and coded data and that all views had been considered in analysis.

Once provisional themes had been identified, the LUPUS UK forum moderator (PH) posed several questions^18,19,20^ to the online community to confirm that emerging themes reflected the wider community views, and to allow the lead researcher (MS) to probe responses in more depth. These questions included: The impact of fatigue and how it feels, the impact of MH/MUS misdiagnoses, and an invitation to discuss positive and negative medical interactions. These questions received between 113 and 197 responses (including researcher questioning) with, for example, the fatigue question eliciting responses from 44 different members. Quotes from the online questioning have not been used in this paper, as they largely replicated and were in agreement with, those in the online questionnaire, thus strengthening validity.

Validity of the findings was also strengthened by member checking^21,22^, where the draft and final results were shared and discussed with multiple members of the lupus/ systemic autoimmune rheumatic disease community to ensure agreement and that analysis was in line with their views. Validity was also strengthened by comparing emerging themes with forum discussions and by examining deviant cases^23^. Further triangulation occurred by the integration of qualitative and quantitative data^24,25^. In this case the qualitative data was complementary to the quantitative data and also allowed individual/combined experiences to be used to add further depth and insight to the quantitative results.

**Supplementary Table S1 Themes and further quotes on identified sub-themes to improve diagnosis and care.**

| **Supplementary Information 2 – Further quotes on identified themes to improve diagnosis and care** |
| --- |
| **Theme 1*: The importance of listening* and *believing patients’ self-reported symptoms***  **Listening to patients**  *‘Really listen to the patient with an open mind. The patient is often the only expert on their body where this particular condition is concerned given that the permutations of presenting symptoms and reactions to medications can be truly unique to that individual’ (*Female, 50s, Scotland)  *‘Listen, listen, listen. If s/he has been treated unfairly prior to diagnosis, it may have a lasting effect which could be misconstrued as depression’* (Female, 70s, England)  **Believing patients’ self-reports of symptoms**  *‘Don’t assume you know what’s wrong then become blinkered by what you believe. Truly see, truly listen, truly believe. When we tell you it’s not all in our heads believe us. We aren’t hypochondriacs and sadly some of us come to believe we are over time, questioning our sanity and actually causing mental illness’* (Female, 30s, Wales)  *‘Just because you can’t identify the pain, doesn’t mean it isn’t there’ (*Female, 40s, Wales)  **Negative test results are rarely felt to be ‘reassuring’ when symptoms remain**  *‘Just because a test is negative doesn’t mean your first words should be ‘good news the test is negative’ If you are at home unable to function, drive, live or work, have to take morphine and can’t go to the loo on your own, being told another test is negative is soul destroying as the Dr looks at you with the ‘we can’t find anything wrong’ look that says ‘go away’* (Female, 40s, England)  **Serological results often do not correspond with patient views of severity of symptoms**  *‘NEVER imply that because bloods look normal, that everything must be OK! Lupus patients often have normal bloods but that doesn’t mean there is nothing going on in the body’* (Female,40s, England)  **Abnormal, atypical and unusual symptoms can be normal, typical and usual for Lupus**  *‘Doctors should try to understand that even if the symptoms and reactions people with Lupus suffer sound unusual it is because the effects of having this illness are unusual. Doctors should listen to the patient with an open mind without trying to put their symptoms into a ‘normal box’. The patient knows best as Professor Graham Hughes says’* (Female, 60s, England)  **Theme 2***:* ***Holistic viewpoint – both in linking symptoms and caring for the whole patient (mental health, wellbeing and quality of life in addition to physical health)***  **Joining the dots**  *‘Don’t treat symptoms independently, treat the patient as a whole, look at the whole picture. Symptoms are so diverse and often seemingly unrelated’* (Female, 70s, England)  **Discussing and assisting with improving quality of life**  *‘I think they should allow more time at appointments for patients to speak about how it affects them day to day. So hospital appointments whilst productive, I feel we go through the motions. Meds check, any change, bloods and goodbye! All apart from my kidney consultant who is amazing and we actually talk about how I have been’* (Female, 40s, England)  **Recognising patients’ need to talk and the value of specialist nurses/ allied health professionals**  *‘I need a lupus nurse or someone with knowledge I could turn to…I don’t want more medication or intervention. I would like someone to talk to who isn’t on a time limit and won’t judge or offer medication I don’t need’* (Female, 50s, England)  *‘I often feel delicate because it’s hard for others to understand the disease. When I go to my consultants I like to open up to them, I cry sometimes and I’m very lucky I can do that…just listening can truly make us feel better’ (*Female, Scotland, age withheld)  **Providing one key physician to ensure the holistic view and effective multi-disciplinary communication**  *‘At one point I was dealing with my GP, a dermatologist, a rheumatologist and a breathing specialist – it all felt very disconnected and I wasn’t sure who was in charge. A named person who can help you make sense of the different people would be invaluable. It’s the whole body that’s affected, Doctors need to communicate with each other to work out the best course of action rather than take their little bits’ (*Female, 30s, Scotland)  **Encouraging peer support**  *‘I think the complexity and abundance of physical symptoms often means that the time and attention (both my own and the Drs) to emotional and coping aspects of SLE gets eclipsed. I have been most impressed by the peer support [facebook/forums] provides, especially in validating and empathising with how this disease FEELS as a whole’* (Female, 40s, England)  **Providing empathy and support in overcoming traumatic diagnostic journeys**  *‘I think Drs would do well to remember that a patient might have gone through psychological trauma in the years leading up to diagnosis, through not being believed or having symptoms dismissed. It’s important to be sensitive to the psychological needs of patients alongside treating the actual lupus’* (Female, 40s, England)  *‘Be aware that many had a difficult health journey and perhaps seen many dismissive doctors before. This affects our ability to present our own cases and may make us appear ‘prickly’ Really we are terrified. A little kindness goes a long way’* (Female, 60s, England)  **Theme 3***:* ***Knowledge – acquisition, exchange and admitting knowledge limitations***  **Support and information sharing at diagnosis**  *‘I feel that I have been left to process this information very much on my own. I did think counselling and treatment would start very quickly but have been left in limbo…I feel very unsupported. Consultants need to understand the impact those few words have on a person, just being told you have lupus and given a leaflet isn’t helpful’ (* Female, 60s, England)  **Respecting the patient’s knowledge**  *‘We frequently know more about our disease than GPs in particular so listen to patients and don’t feel threatened by their knowledge or suggestions’* (Male, 20s, Scotland)  **Empowering patients**  *‘Treat disease management as a partnership. Autoimmunity is very complex and needs constant monitoring by the patient. For that they need to be empowered and feel confident’* (Female, 50s, England)  **Admitting limits of knowledge, referring and working with lupus specialists**  *‘If Lupus is suspected give an appointment at a centre of excellence where tests can be carried out by people who are experts with any recommendations to be followed locally by GP and rheumatologist. A plan in place if flares happen or need access to urgent medications. For local rheumatologists who have expertise in other areas like RA to be open to working with centres of excellence and not feel threatened by their recommendations’* (Female, 50s, Wales)  *‘I asked for 5 referrals out of area to see various doctors, mostly lupus specialists…every time I was told that there was no point in applying, it would be denied. We desperately need help here’.* (Female, 40s, Wales)  **Constantly acquiring knowledge**  ‘*The support I have received from my GP has been great. I accept that he won’t have detailed training or knowledge of lupus but he’s been honest and will find out any questions he can’t answer’ (*Female, 50s, England) |
